# Establishing a standardized biorepository across 3 clinical and translational science center–affiliated veterinary institutions is feasible and productive

**DOI:** 10.2460/ajvr.26.02.0045

**Published:** 2026-05-11

**Authors:** LaTasha K. Crawford, Molly E. Church, K. A. Boudreaux, J. N. Lapin, Lauryn A. Hahn, Leslie J. Solis, Rachael Wilson, Henrik Zetterberg, Fernanda Catacci Guimaraes, Sam Shen, Ryan G. Toedebusch, Karen Vernau, Adrien Dupanloup, Wojciech Panek, Starr Cameron, Christine M. Toedebusch

**Affiliations:** 1 Department of Pathobiological Sciences, School of Veterinary Medicine, University of Wisconsin-Madison, Madison, WI; 2 NeuroBiobank, School of Veterinary Medicine, University of Wisconsin-Madison, Madison, WI; 3 Department of Pathobiology, School of Veterinary Medicine, University of Pennsylvania, Philadelphia, PA; 4 Office of Professional Education, School of Veterinary Medicine, University of California-Davis, Davis, CA; 5 Department of Pathobiology, School of Veterinary Medicine, University of Pennsylvania, Philadelphia, PA; 6 Clinical and Translational Science Center, University of California-Davis, Davis, CA; 7 Department of Pathology and Laboratory Medicine, School of Medicine and Public Health, University of Wisconsin-Madison, Madison, WI; 8 Wisconsin Alzheimer’s Disease Research Center, School of Medicine and Public Health, University of Wisconsin-Madison, Madison, WI; 9 Department of Psychiatry and Neurochemistry, Institute of Neuroscience and Physiology, The Sahlgrenska Academy at the University of Gothenburg, Mölndal, Sweden; 10 Clinical Neurochemistry Laboratory, Sahlgrenska University Hospital, Mölndal, Sweden; 11 Veterinary Neurology Biobank, School of Veterinary Medicine, University of California-Davis, Davis, CA; 12 Department of Surgical and Radiological Sciences, School of Veterinary Medicine, University of California-Davis, Davis, CA; 13 Department of Molecular Biosciences, School of Veterinary Medicine, University of California-Davis, Davis, CA; 14 Department of Clinical Sciences & Advanced Medicine, School of Veterinary Medicine, University of Pennsylvania, Philadelphia, PA; 15 Department of Medical Sciences, School of Veterinary Medicine, University of Wisconsin-Madison, Madison, WI

**Keywords:** biorepository, biomarkers, Clinical and Translational Science Award, Freezerworks, fitness-for-purpose

## Abstract

**Objective:**

To assess faculty interest in veterinary biospecimens, develop a standardized multi-institutional biological fluid repository across 3 veterinary institutions, and assess quality and fitness for purpose of archived samples.

**Methods:**

We conducted a prospective multi-institutional biobanking and feasibility study from 2022 through 2025. Faculty from each institution were administered a voluntary and anonymous survey via email, and responses were recorded. Biological fluid specimens were collected and archived from the Neurology and Neurosurgery Services of each participating institution. A shared database (Freezerworks) was utilized by all 3 institutions to annotate each sample with clinical data and record the storage location. Selected serum (n = 12), CSF (n = 12), and urine (n = 9) samples were assessed for quality control and fitness for purpose by measuring concentrations of putative biomarkers via ELISAs and single-molecule array, respectively.

**Results:**

Faculty surveys indicated widespread support for centralized institutional biobanking, with 94 of 170 respondents (55%) in favor of a combination of institutional and investigator financial support. Over 2,500 biological specimens from dogs and cats with neurological disease were collected during the 3-year study period. Sample quality across serum, urine, and CSF was consistent across institutions. Moreover, serum samples from randomly selected dogs with divergent diagnoses yielded robust and discriminatory concentrations of putative neurodegenerative disease proteins.

**Conclusions:**

Multi-institutional veterinary biobanking is feasible and productive, providing investigators with access to clinically annotated, high-quality biospecimens.

**Clinical Relevance:**

A common repository registry, wherein investigators have reciprocal access to standardized biospecimens, will connect investigators from all domains to promote rigorous, well-powered multicenter studies.

In 2002, the National Cancer Institute surveyed the scientific community and learned that the limiting factor for translational cancer research was a lack of standardized, high-quality biospecimens with well-annotated clinical data. In response, the National Cancer Institute Office of Biorepositories and Biospecimen Research developed evidence-based standardized protocols for the collection, processing, and annotation of biospecimens and established the Cancer Human Biobank for specimen archiving and distribution. This is just a single example of how the practice of collection, preservation, and accessibility of biological samples and associated data^1^ has become an organized science in human biobanking, with large-scale infrastructure across many platforms. Standardized sample processing, data annotation, and ease of sample distribution to the scientific community have connected basic, translational, and clinical research with clinical practice,^2^ resulting in collaborative research, reduced cost, and improved care of cancer patients.^3^ Veterinary biobanking has a similar potential for widespread impact, but progress lags behind what has been done in human biobanking. This resource gap is especially pertinent for biological samples from veterinary patients with neurological diseases that lack antemortem diagnostic biomarkers and have high translational value.

Veterinary research serves as the interface of basic science and animal and human health that is fundamental to the pursuit of One Health.^4^ Thus, veterinary biospecimens have tremendous potential to accelerate human and animal health research. In particular, the dog has become increasingly recognized as a model for several human conditions, including numerous types of cancer,^5^ epilepsy,^6^ and aging.^7^ With routine access to a wide array of biological samples, veterinary biobanks have the unique potential to collect more numerous samples with translational relevance that can be complementary to human biobanks.^1^ Moreover, as companion animals age rapidly relative to humans,^8^ longitudinal sample collection over the animal’s lifetime and throughout the course of a chronic disease is more feasible.

Despite their inherent value, there are critical barriers to successful, sustainable biobanks. While many individuals have assembled veterinary biospecimen collections, there is a broad lack of standardized protocols for collection and archiving.^1^ Variations in collection, sample handling, and storage procedures are a leading driver of scientific error and lack of data reproducibility, particularly within biomarker development,^9^ highlighting the necessity for optimized sample-handling protocols. Additionally, lack of shared access to biospecimens^1^ impedes successful, sustainable veterinary biobanking and utilization for translational research. Currently, many veterinary biobanks are individual- or institution-led entities, with little knowledge of similar or complementary collections with other investigators. As a result, the burden of operational and financial sustainability relies solely on the individual, which may preclude comprehensive and lasting specimen collection. Importantly, lack of shared access perpetuates underpowered studies, lengthens the timeline for sample acquisition, and deters the study of uncommon conditions, thus mitigating the power of translational veterinary research in the pursuit of One Health.

To address the lack of standardized protocols and shared access to biospecimens in veterinary biobanking, we developed a shared repository of well-annotated dog and cat biological fluid specimens across 3 veterinary teaching hospitals in the US: the University of California-Davis (UC-Davis), the University of Wisconsin-Madison (UW-Madison), and the University of Pennsylvania (UPenn). Key performance indicators of successful biobanks include quality, activity, scientific productivity, and visibility.^10,11^ Therefore, the objectives of this study were to (1) assess interest and current unmet needs for access to veterinary biospecimens of investigators across these 3 institutions, (2) develop standardized biological fluid collection and storage conditions across multiple veterinary institutions, (3) determine biospecimen quality of samples archived through the shared repository as recommended by the International Society for Biological and Environmental Repositories (ISBER),^8^ and (4) evaluate biospecimen fitness for purpose.

## Methods

### Assessment of faculty needs for veterinary biospecimens across institutions

We surveyed all clinical and research faculty from the veterinary schools of UC-Davis, UW-Madison, and UPenn. Thirteen questions were developed to assess general interest in, or ongoing activities related to, the utilization of biological samples from domestic animal species. The questions were populated into a Qualtrics survey platform (Supplementary Material S1) and dispersed to all faculty via electronic mail; this study was determined to be exempt by the institutional review board. Faculty were given 4 weeks to submit their responses.

### Development of standardized, multi-institutional biological fluid repository

We collaborated with the UC-Davis Clinical and Translational Science Center and their Biobanking Operations Specialist (LJS) to develop a customized database for specimen annotation and management using Freezerworks Laboratory Information Management System software, centrally hosted at UC-Davis. This system enables efficient and reliable tracking of longitudinal biospecimen collections across all 3 institutional sites. Using the UC-Davis Neurology Biorepository as the foundational template, we configured shared data collection fields, standardized data entry forms, and a virtual freezer layout for each additional site. This design allows each institution’s inventory to be visible and searchable across sites with read-only access, maintaining data integrity. In addition, a designated restricted freezer section was created for each institution to organize samples specific to investigator-initiated projects that were reserved separately from shared-access biospecimens.

Individual animals were referred to as “participants” within the Freezerworks platform. Animal signalment and disease information were created for each participant in the Freezerworks database by investigators at each institution. Within a participant’s record, the date and time of collection and storage were recorded as well as the precise location in the freezer. The Freezerworks platform links all serial collections and multiple sample types from a single participant for ease of organization. Disease diagnoses were annotated with terminology that was used within the medical record system of each institution and standardized across institutions.

Samples were collected following clinical trials review board (UC-Davis) and IACUC approval at each respective institution (UC-Davis, No. 24266; UW-Madison, No. V006548; UPenn, No. 807245). At each institution, client consent was obtained by the Neurology clinician (faculty and residents). Client consent forms were tailored specifically to each institution’s IACUC standards. From 2022 through 2025, samples were collected from dogs and cats presenting to the Veterinary Neurology & Neurosurgery Services at each of the 3 institutions that met the following inclusion criteria: (1) signed client informed consent, (2) well-characterized neurological signs with advanced neurodiagnostics enabling a clinical and/or definitive diagnosis, and (3) adequate sample yield and same-day sample processing.

Whole blood, urine, and/or cerebrospinal fluid (CSF) were collected at each institution, when possible, to ensure a comprehensive biofluid profile while maintaining a feasible workload on staff throughout the process of collection and storage. A single sample-handling protocol, in line with the ISBER^8^ best practices,^12^ was utilized across participating institutions (Supplementary Material S2). Kits for each fluid type were premade to facilitate ease of collection by staff. T310 Cryovials (Simport) or CryoPure polypropylene tubes with external threads (Sarstedt), a preferred vial for long-term biospecimen storage,^9^ were labeled with Freezerworks labels containing a barcode for fast tracking, an aliquot identification number, and a unique participant identification number for all samples within a single kit. Lids were color coded according to sample type for ease of identification by staff (eg, green, CSF; red, serum; yellow, urine). Three vials were labeled for each kit and placed in zip-lock bags containing a blank outer label with spaces designated for patient stickers, patient medical information (eg, presumptive diagnosis), and sample information (eg, time of collection, time placed in freezer).

Samples were collected according to standard veterinary practices by trained hospital personnel. The date, time, method (eg, venipuncture), and site of collection (eg, cerebellomedullary cistern for CSF collection) were recorded into a hand-written sample log and/or the individual kits. Samples were aliquoted to preserve sample integrity and enhance sample distribution (urine and serum, 500 μL/vial; CSF, 200 μL/vial). Specimens were stored at −20 °C within 1 hour of collection and transferred to −80 °C for long-term storage within 5 days of collection. The date and time of storage at −20 and −80 °C transfer were recorded. Annotated sample information was entered into Freezerworks upon long-term storage.

Each sample type was collected and processed as follows:

Serum—Whole blood (1 to 2 mL) was collected by venipuncture or intravascular catheter aspiration and placed in serum separator tubes. After clot formation, tubes were centrifuged (2,236 X *g* for 10 minutes), and the serum was aspirated, aliquoted, and stored at −20 °C.

Cerebrospinal fluid—Concurrent with CSF collection as clinically indicated, clinicians would secure additional samples. Cerebrospinal fluid (up to 1 mL) was collected with a spinal needle from the cerebellomedullary cistern or the lumbar subarachnoid space directly into cryovials (ie, T310 Cryovials or CryoPure polypropylene tubes), or into a red-top tubes and aliquoted into cryovials, and stored at −20 °C.

Urine—Urine (1 to 2 mL) was collected by free catch, cystocentesis, or closed collection systems, aliquoted, and stored at −20 °C.

Deoxyribonucleic acid—Whole blood was collected by venipuncture or intravascular catheter aspiration, placed in EDTA tubes, and stored at 4 °C. The white blood cell (WBC) pellet was isolated as previously described.^10^ Briefly, 1 mL whole blood was aspirated from the EDTA tube, mixed with red blood cell (RBC) lysis solution (1:3 ratio of whole blood to RBC lysis solution), and incubated at room temperature (RT) for 10 minutes. Samples were centrifuged (500 X *g* for 5 minutes) at RT, and the supernatant was discarded. The process was repeated 1 to 3 times as needed until there was no RBC pellet visible. The WBC pellet was flash frozen in liquid nitrogen or dry ice before transfer to −80 °C for long-term storage.

### Evaluation of archived biospecimen sample quality

To evaluate prolonged RT exposure and storage conditions across biological fluid type, we selected canine participants from each institution’s archive with a diagnosis of idiopathic epilepsy. Inclusion criteria for participant sample selection were (1) tier 1 diagnosis of idiopathic epilepsy according to the International Veterinary Epilepsy Task Force,^13,14^ (2) normal brain MRI and CSF analysis, (3) no systemic comorbidities or concurrent neurological disease, and (4) availability of at least 1 aliquot each of serum, urine, and CSF that was collected and archived on the same hospital visit. The shared Freezerworks network allowed for a single investigator (CMT) to query each participating institution’s archive and select cases meeting the inclusion criteria; cases were confirmed by each study investigator. Samples from UW-Madison and UPenn were sent to UC-Davis on dry ice and stored at UC-Davis at −80 °C until analysis.

We utilized commercially available, canine-specific ELISAs to determine biofluid-specific concentrations of quality control biomarkers recommended by the ISBER in human biobanking. Specimens from each institution were assayed for recommended quality control biomarkers as follows: serum, soluble CD40 ligand (CD40L^15^; catalog No. ELC-CD40L; RayBiotech); urine, tumor necrosis factor–α (TNF-α^12^; catalog No. ELC-TNFa; RayBiotech); and CSF, cystatin-C (catalog No. EC2RB; Invitrogen Corp). It should be noted that the ISBER^8^ has indicated that cystatin-C requires further validation for CSF quality control,^16^ but alternatives for CSF are lacking.^16^ We included analysis of cystatin-C to evaluate for reproducibility across institutional biospecimens.

Protein concentrations of each target were measured with each respective ELISA kit according to the manufacturer’s instructions. Briefly, samples were plated undiluted (100 μL sample/well), washed with a microplate washer (BioRad 1575 ImmunoWash; BioRad Laboratories), and had their absorbance (450 nm) read using an absorbance microplate reader (800 TS; BioTek). Standards and all samples were plated and evaluated in triplicate.

### Fitness for purpose: neurodegenerative disease biomarker pilot study

To evaluate the fitness for purpose of archived samples from neurological veterinary patients, we evaluated the concentrations of putative biomarkers of human neurodegenerative disease in canine serum. Cases were randomly selected and included 1 each of the following presumptive disease processes: orbital blastomycosis with intracranial extension, high-grade glioma, and aggressive behavior without structural brain disease. Biomarker assays were performed under the direction of Dr. Zetterberg at the Wisconsin Alzheimer’s Disease Research Center. Frozen canine serum samples from UW-Madison were submitted to the Alzheimer’s Disease Research Center Biomarker Lab and analyzed using the HD-X automated high-throughput immunoassay platform (Quanterix) as previously utilized in canine biofluids.^17–21^ Biomarkers were measured using the single-molecule array (SIMOA) Human Neurology 4-Plex E assay (Quanterix) for detection of amyloid β (Aβ) 1–42 (Aβ42) and Aβ 1–40 (Aβ40), proteins related to amyloid plaque aggregation, and glial fibrillary acidic protein (GFAP) and neurofilament light (NfL), markers of neuroinflammation and neurodegeneration, respectively.

### Statistical analysis

Statistics were performed for ELISA data in this study. For each biomarker, concentrations were tested for normality using the Shapiro-Wilk test and compared across institutions by Kruskal-Wallis ANOVA on ranks with post hoc Dunn multiple-comparisons test. Data are presented as median with IQR.

## Results

### Faculty surveys indicate widespread support for centralized institutional biobanking

**Figure 1 —:**
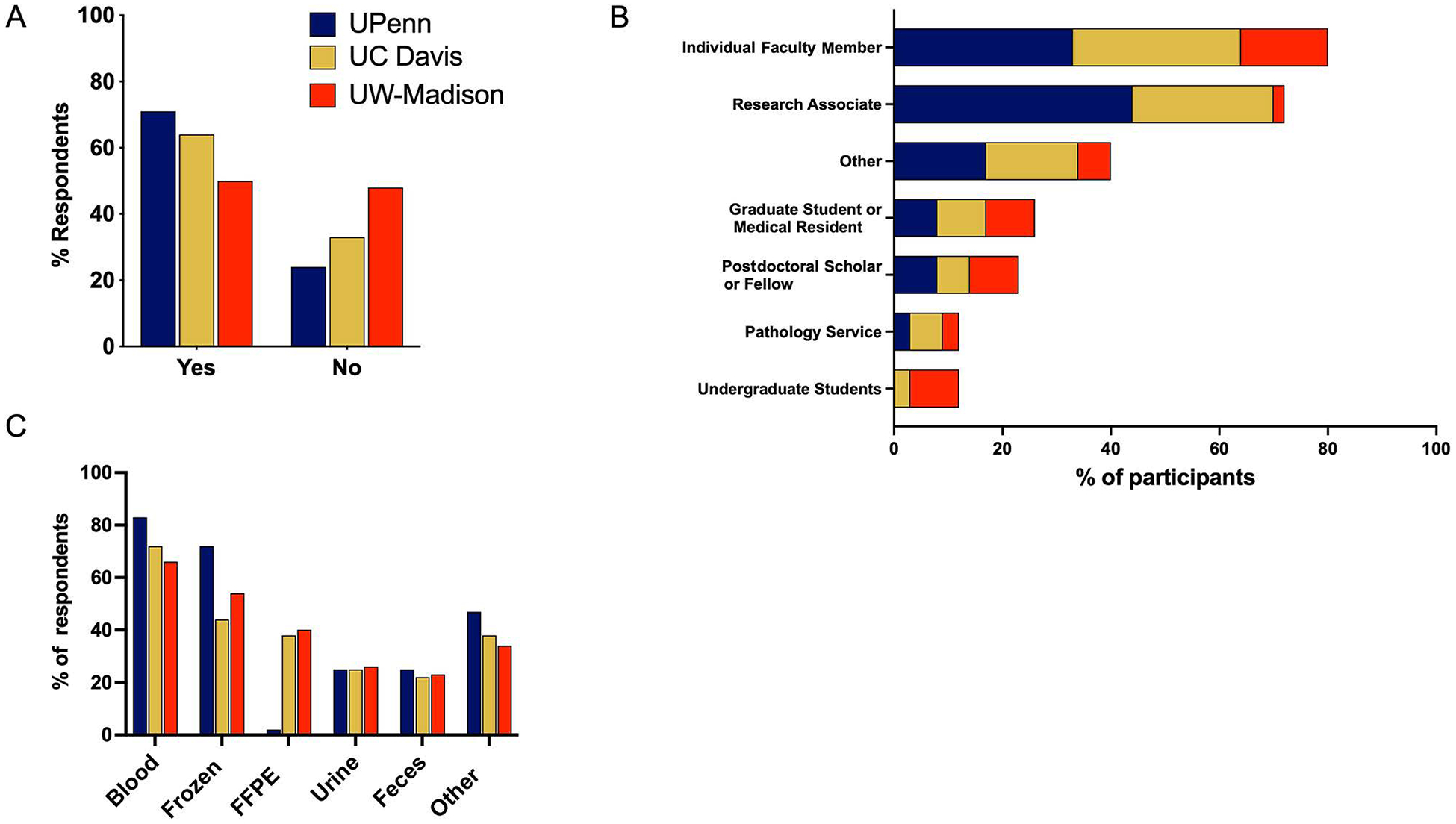
Veterinary biospecimens are important to faculty research across 3 veterinary teaching hospitals. A—Of the survey responders, 70%, 64%, and 50% of clinical and research faculty from the University of Pennsylvania (UPenn), the University of California-Davis (UC-Davis), and the University of Wisconsin-Madison (UW-Madison) currently utilize biospecimens in their research program. B—Sample collection and archiving is most performed by the individual faculty investigator or research associates, such as laboratory technicians and project scientists, at all 3 institutions. C—Blood and fresh frozen tissue are the most collected biospecimens. Data are expressed as a percentage of all survey respondents of each institution; navy blue bars represent UPenn, gold bars represent UC-Davis, and red bars represent UW-Madison. FFPE = Formalin-fixed, paraffin-embedded.

One hundred seventy (41%) faculty members across 3 institutions responded to the online survey (UPenn, n = 51 of 115; UC-Davis, 55 of 300; UW-Madison, 64 of 100). Respondents represented a wide range of positions, including clinical, teaching, and research track instructors and faculty across the assistant, associate, and full professor levels. An average of 61% of respondents across all institutions currently utilize biological samples from veterinary species (UPenn, n = 36 [71%]; UC-Davis, 35 [64%]; UW-Madison, 32 [50%]; Figure 1). Many survey respondents across all institutions indicated that they themselves are responsible for collecting samples for their project (UPenn, n = 17 [33%]; UC-Davis, 17 [31%]; UW-Madison, 10 [16%]). Research associates are also widely utilized across institutions (UPenn, n = 16 [31%]; UC-Davis, 9 [16%]; UW-Madison, 8 [13%]). Respondents from all institutions indicated that they utilize support from trainees, including undergraduate, graduate, and veterinary students; veterinary medical residents; and postdoctoral scholars. A small number of respondents from all institutions indicated that they utilize their institution’s anatomic pathology service (UPenn, n = 1 [2%]; UC-Davis, 2 [4%]; UW-Madison, 1 [2%]). Blood and fresh frozen tissue were the most common sample types collected, although respondents from each institution indicated collection of a broad range of sample types. Cerebrospinal fluid, synovial fluid, and oral swabs were commonly identified as “other” sample types collected.

**Figure 2 —:**
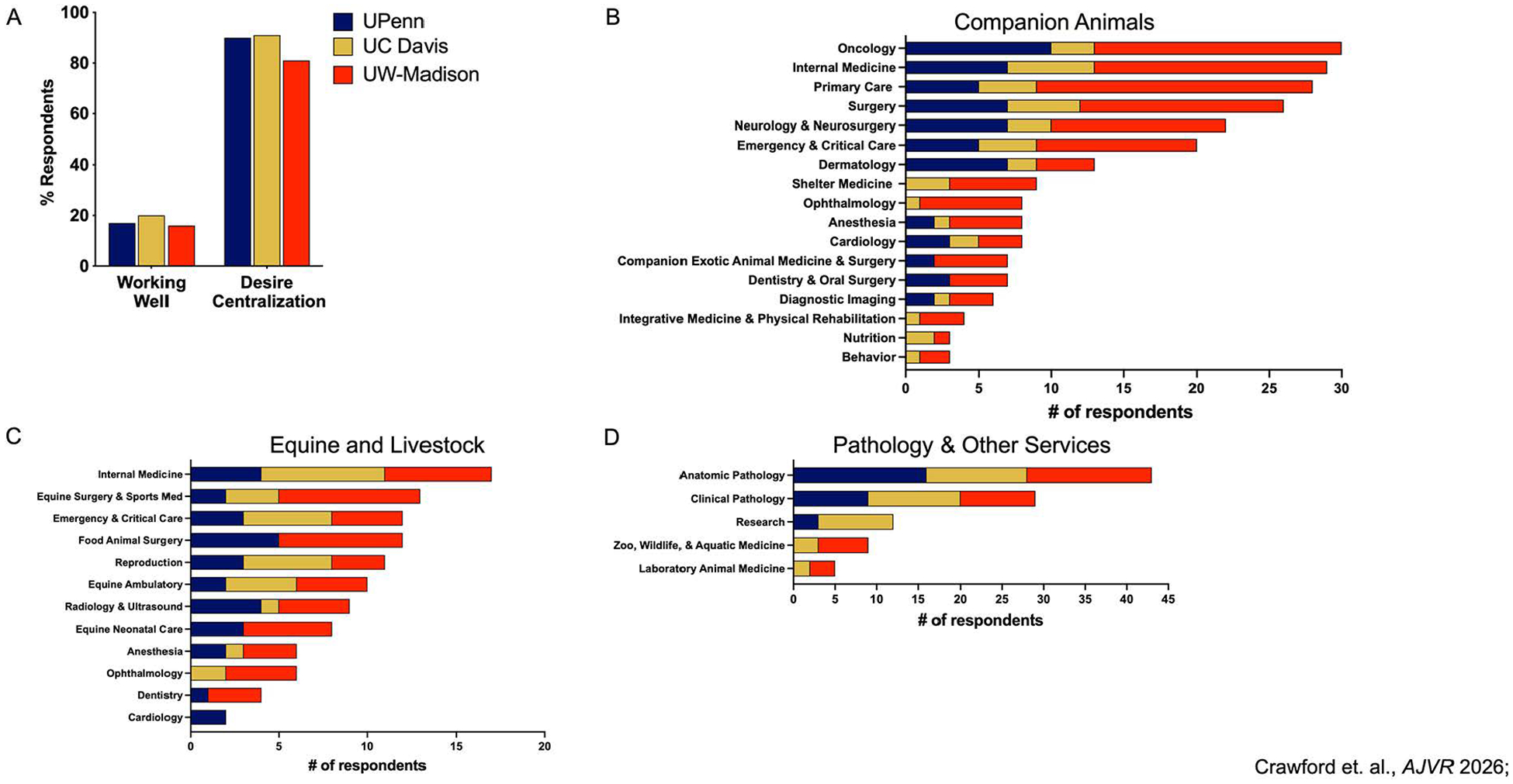
A centralized biobank is unanimously desired to improve faculty research productivity. A—Approximately 20% of survey responders say their current biospecimen collection method is working well. More than 80% of survey responders responded “yes” when asked if they would be interested in a centralized biobank within their institution. B—Biospecimens desired from Companion Small-Animal and Exotic Services included Oncology, Internal Medicine, Primary Care, and Surgery. C—Respondents indicated a desire to procure biospecimens from a variety of Equine and Livestock Services as well as (D) Anatomic Pathology and other non–client-facing services. Data are expressed as a percentage of all survey respondents of each institution for A, C, D, and E; navy blue bars represent UPenn, gold bars represent UC-Davis, and red bars represent UW-Madison.

A strong majority of respondents from UPenn (n = 43 [85%]), UC-Davis (50 [90%]), and UW-Madison (51 [80%]) indicated that they would be interested in a centralized biobank within their own institution (Figure 2). When asked where financial support should come from for a centralized biobank, more than 50% of respondents indicated that financial support should come from a combination of administrative support and investigator-initiated grant funding, whereas 37% of respondents indicated that a centralized biobank should be fully funded by institutional administrative support. Respondents indicated a desire for animal biospecimens across nearly every domain in veterinary medicine. Investigators seeking biospecimens from companion animals (including exotic species) were particularly interested in patients receiving treatment from Oncology, Internal Medicine, Primary Care, Surgery, and Neurology & Neurosurgery Services. Investigators seeking biospecimens from equine and livestock patients were particularly interested in patients receiving treatment from Internal Medicine, Equine and Food Animal Surgery, Emergency & Critical Care, and Reproduction. More than 20% of respondents from each institution (UPenn, n = 16 of 51; UC-Davis, 12 of 55; UW-Madison, 15 of 64) indicated interest in samples from Anatomic Pathology.

### Biological fluid collection was feasible and productive across institutions

**Figure 3 —:**
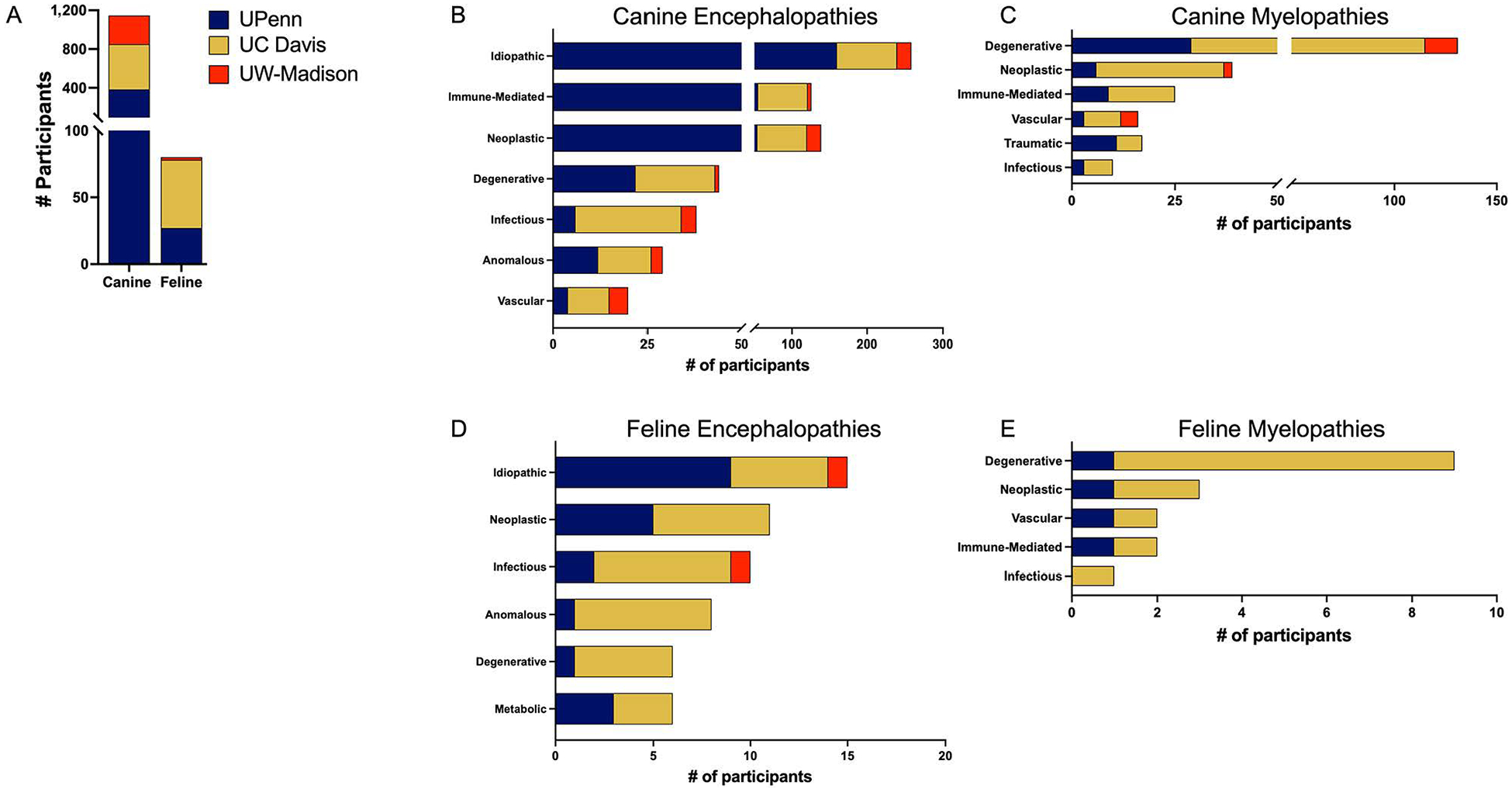
A multi-institutional veterinary biobank is feasible and productive. A—From 2022 through 2025, biospecimens were collected from 931 unique canine and 80 unique feline. B—Encephalopathic disease categories affecting canine participants that had biospecimens collected most included idiopathic (n = 258), immune-mediated (n = 126), and neoplastic (n = 139) diseases. C—Myelopathic disease categories affecting canine participants that had biospecimens collected most included degenerative (n = 131), neoplastic (n = 39), and immune-mediated (n = 25) diseases. D—Encephalopathic disease categories affecting feline participants that had biospecimens collected most included idiopathic (n = 15), neoplastic (n = 11), and infectious (n = 10) diseases. E—The most common myelopathic disease category affecting feline participants that had biospecimens collected included degenerative disease (n = 9). Data are expressed as a percentage of all survey respondents of each institution; navy blue bars represent UPenn, gold bars represent UC-Davis, and red bars represent UW-Madison.

Each institution successfully established and maintained the infrastructure for biobanking of biological fluids from veterinary patients with neurological disease throughout the 3-year study period. Across all 3 institutions, biospecimens were collected from 931 dogs and 80 cats, referred to as “participants” (Figure 3). Of canine participants with encephalopathies, idiopathic (n = 258), immune-mediated (n = 126), and neoplastic (n = 139) etiologies were most common. Tier 1 and 2 idiopathic epilepsy and meningoencephalitis of unknown origin were the most common diagnoses for idiopathic and immune-mediated diseases, respectively. Neoplastic diseases varied, with collection from patients with meningioma and oligodendroglioma primary brain tumors most common. Myelopathic disease categories affecting canine participants that had biospecimens collected most included degenerative (n = 131), neoplastic (n = 39), and immune-mediated (n = 25) etiologies. Participants with type 1 intervertebral disk disease were most common.

Across institutions, feline participants were less common than canine participants. However, each biobank was able to collect from feline participants with a variety of disease etiologies. Of feline participants with encephalopathic disease, idiopathic (n = 15), neoplastic (n = 11), and infectious (n = 10) etiologies were most common (Figure 3). Tier 1 and 2 idiopathic epilepsy and meningiomas were the most common diagnoses for idiopathic and neoplastic diseases, respectively. Five of 9 cases of infectious encephalopathy were secondary to otitis media/interna with intracranial extension. The most common myelopathic disease category affecting feline participants that had biospecimens collected included degenerative disease (n = 9), with type 1 intervertebral disk disease the most common.

**Figure 4 —:**
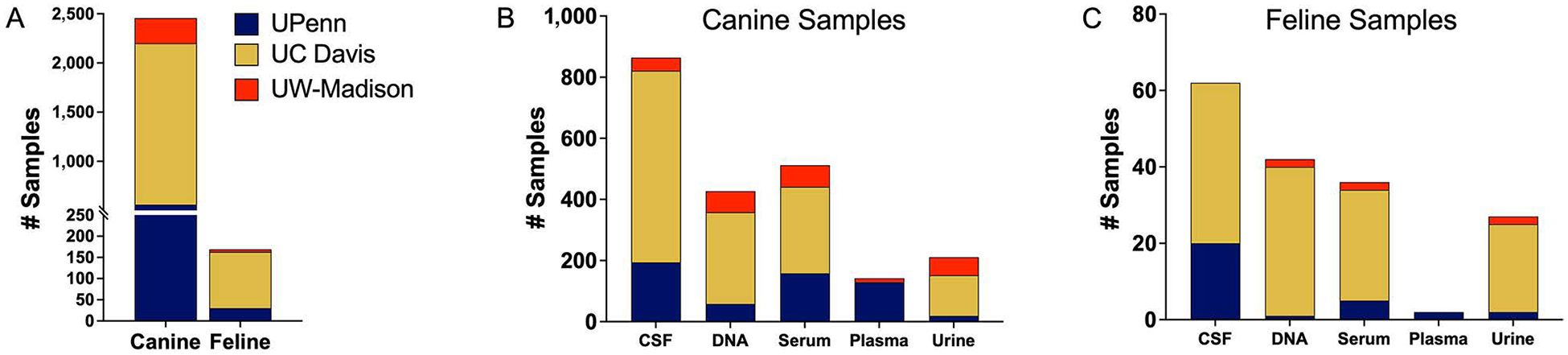
A—Biospecimens from canine participants were the most abundant (n = 2,156), with (B) CSF (n = 864) and serum (n = 512) the most commonly collected. C—There were 169 samples collected from feline participants, with (D) CSF (n = 62) and DNA (n = 42) the most commonly collected.

A total of 2,325 biospecimens were collected during the 3-year study period. A total of 2,156 samples were from canine participants (UPenn, n = 558; UC-Davis, n = 1,343; UW-Madison, n = 255; Figure 4), with CSF (n = 864) and serum (n = 512) the most abundant sample types collected. There were 169 samples collected from feline participants during the study period. CSF (n = 62) and DNA (n = 42) were the most abundant sample types collected.

### Sample quality across fluid type was consistent across institutions

Twelve cases (n = 4/institution) met the inclusion criteria for quality control evaluation (Supplementary Table S1). Each institution provided 4 serum and CSF samples each. University of Wisconsin-Madison and UC-Davis provided 4 urine samples; UPenn had a single urine sample. The cases represented dog breeds including Labrador Retriever (n = 3), mixed (n = 3), Golden Retriever (n = 2), Australian Shepherd (n = 1), Belgian Malinois (n = 1), and German Shorthaired Pointer (n = 1). The median dog age at the time of sample collection was 4.5 years (range, 3 to 9 years). Most dogs were castrated males (n = 9 [75%]). The sexes of the remaining 3 dogs were intact male (n = 1), intact female (n = 1), and spayed female (n = 1).

**Figure 5 —:**
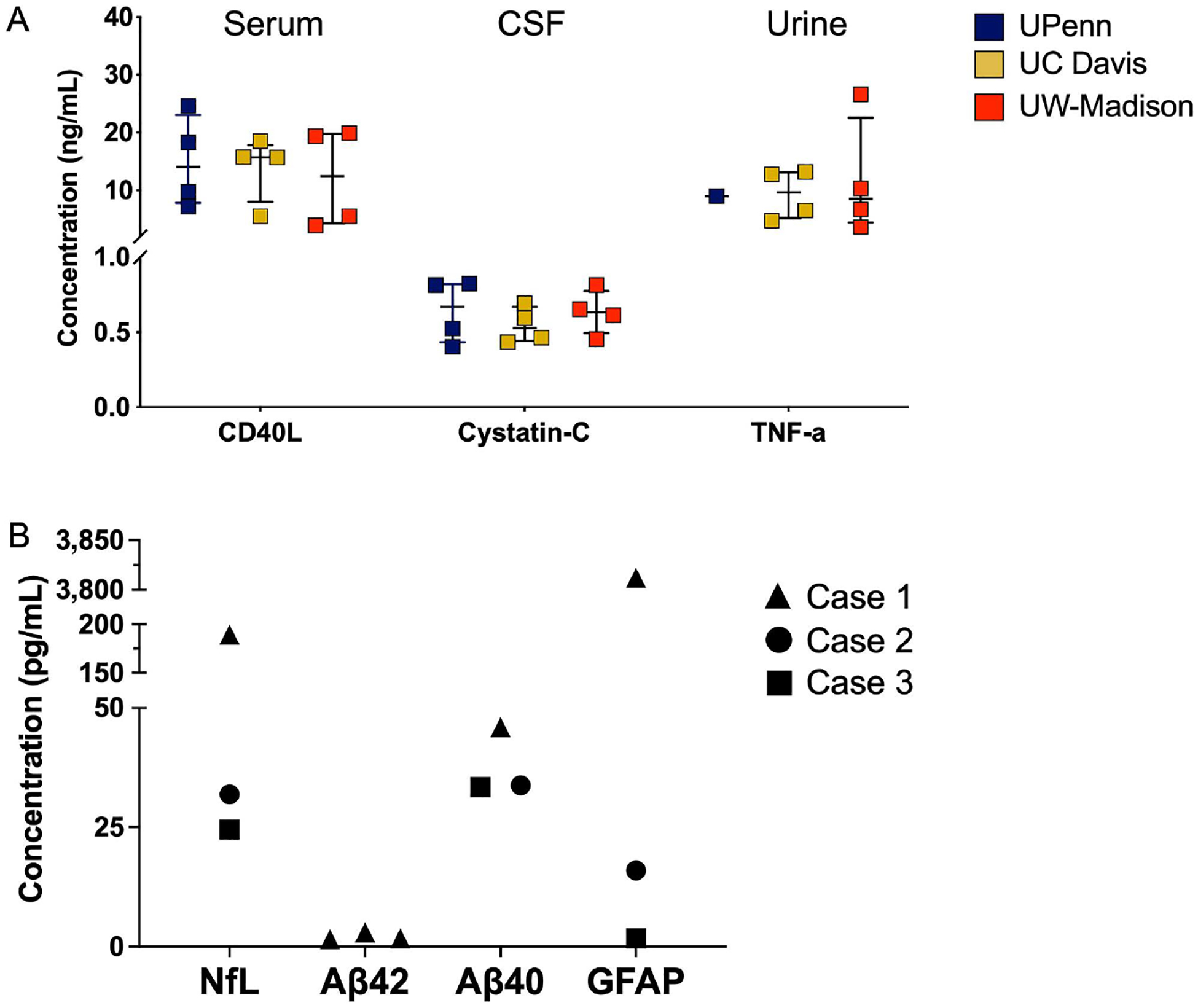
Biospecimens procured through a multi-institutional biobanking effort produce high-quality samples that demonstrate fitness for purpose. A—Scatter-dot plot of concentrations of selected quality control biomarkers measured via commercially available ELISA kits in serum, CSF, and urine from dogs with tier 1 diagnosed idiopathic epilepsy (n = 4 dogs/institution). The CD40L (serum; *P* = .9884), cystatin-C (CSF; *P* = .7269), and tumor necrosis factor–α (TNF-α; urine; *P* = .9500) concentrations were robust and similar across UPenn, UC-Davis, and UW-Madison. Each dot represents an individual patient; lines represent the group median with 95% CI. Navy blue boxes represent samples from UPenn, gold boxes represent samples from UC-Davis, and red boxes represent samples from UW-Madison. B—Single-molecule array analysis using the Human Neurology 4-Plex E panel (Quanterix) demonstrated robust detection of putative biomarkers of neurodegenerative disease across 3 patient serum samples randomly selected from the UW-Madison biobank. Aβ = Amyloid β. CD40L = CD40 ligand. GFAP = Glial fibrillary acidic protein. NfL = Neurofilament light.

Quality control biomarkers were assessed in biofluid samples from all 3 sites using commercially available ELISAs (Figure 5). Median serum CD40L concentrations were similar across samples from each site (UPenn, 14.96 ng/mL [IQR, 7.84 to 23.03 ng/mL]; UC-Davis, 15.74 ng/mL [IQR, 15.65 to 18.5 ng/mL]; UW-Madison, 15.74 ng/mL [IQR, 5.5 to 19.35 ng/mL]; *P* = .9884). Median CSF cystatin-C concentrations were similar across samples from each site (UPenn, 0.67 ng/mL [IQR, 0.43 to 0.82 ng/mL]; UC-Davis, 0.53 ng/mL [IQR, 0.44 to 0.67 pg/mL]; UW-Madison, 0.63 ng/mL [IQR, 0.49 to 0.77 ng/mL]; *P* = .7269). Median urine TNF-α concentrations were similar across samples from UC-Davis (12.76 ng/mL [IQR, 4.76 to 13.16 ng/mL]) and UW-Madison (8.52 ng/mL [IQR, 4.4 to 22.5 ng/mL]; *P* = .9500). University of Pennsylvania had a single urine sample with a TNF-α concentration of 9.0 ng/mL.

### Serum samples demonstrate robust fitness for purpose

To further validate the quality of our samples and evaluate fitness for purpose, we utilized SIMOA assays on serum samples from 3 randomly selected dogs with divergent clinical diagnoses. All biomarkers in the SIMOA Neurology 4-Plex E panel (Aβ40, Aβ42, GFAP, and NfL) were detected in every serum sample (Figure 5). As expected, we observed considerable variability in protein concentrations of each neurodegenerative disease–associated biomarker across patient samples. The median serum Aβ40 concentration was 37.5 pg/mL (range, 33.0 to 46.0 pg/mL). The median serum Aβ42 concentration was 1.8 pg/mL (range, 1.6 to 3.0 pg/mL). Neurofilament light and GFAP concentrations demonstrated marked variability across patient samples, with median values of 31.9 pg/mL (range, 24.5 to 189.5 pg/mL) and 16.0 pg/mL (range, 1.8 to 3,812.0 pg/mL), respectively. Moreover, we found that serum protein concentrations were consistent between samples undergoing a single freeze-thaw cycle and samples with multiple (n = 2 to 3) freeze-thaw cycles (Supplementary Figure S1).

## Discussion

The objectives of this study were to (1) evaluate investigator interest and unmet needs related to veterinary biospecimen access across 3 veterinary schools, (2) develop a standardized multi-institution biological fluid collection and archival methodology, and (3) determine biospecimen quality and fitness for purpose of samples archived through the shared repository. We found that most faculty members surveyed across 3 institutions were interested in collecting and utilizing biospecimens from veterinary species and were in favor of a centralized biobanking effort. Over a 3-year period, collective archiving across the 3 institutions yielded more than 2,500 biological specimens from dogs and cats with neurological disease and demonstrated consistent sample quality and fitness of purpose.

The survey results indicated that biological specimens from veterinary patients are a critical resource for faculty research, with over 50% of respondents currently utilizing samples. However, 20% to 30% of faculty from each institution are the primary people responsible for sample collection, processing, and archiving. Across veterinary and human biobanks, the time and effort required to establish and maintain biospecimen collections pose a significant barrier for many investigators. This pilot study focused on the collection of samples from patients presenting to the Neurology Services at each of the participating institutions, representing the collection of more than 800 samples collected each year. Expansion to other services, which many clinical and research faculty expressed their desire for, would require more coordination and additional personnel. Consistent with these challenges, more than 50% of respondents expressed support for the development of a centralized biorepository, supported through a combination of investigator-initiated funding and institutional investment. A centralized biorepository with dedicated personnel to aid in collection, accessioning, processing, and archiving of samples would streamline sample collection and management, creating a robust, shared resource for investigators across Clinical and Translational Science Award (CTSA)-affiliated institutions.

The existing biobanking protocols, standard operating procedures, consent form templates, and workflows developed at UC-Davis Neurology Biorepository in line with the ISBER best practices were disseminated to the site principal investigator at UW-Madison (LKC) and UPenn (MEC). Because each institution lacked dedicated biobank staff, sample collection relied heavily upon Neurology clinicians and staff during routine clinical practice. We found that engagement of the clinical team was enhanced through collaborative planning meetings and consistent feedback and response from the clinical team and study investigators. For example, incorporating study consent forms into the initial client paperwork approving diagnostics, storing premade collection kits in convenient locations, and providing easy access to short-term 4 and −20 °C sample storage were key to streamlining sample acquisition and storage.

The practical utility of biobanking infrastructures is influenced by the accessibility and reliability of shared data management systems. Inconsistent access to centralized software platforms may hinder timely data entry and retrieval as well as intrainstitutional and crossinstitutional collaboration. Freezerworks was made available to study investigators through the UC-Davis Clinical and Translational Science Center, which licenses and supports the platform as part of its CTSA-funded research infrastructure. Access was extended to collaborating CTSA-affiliated institutions, including UPenn and UW-Madison. Investigators found Freezerworks to be user friendly and easily customizable to capture relevant patient information for each study. For this study, investigators annotated samples with the patient signalment, disease type, and disease diagnosis. The date and time of collection and storage were recorded for each sample as well as a detailed entry of the precise sample location. To facilitate ease of interinstitution sample searching, diagnostic categories (eg, idiopathic encephalopathy) and diagnoses (tier 1 idiopathic epilepsy) were standardized across institutions. Investigators found that data entry was straightforward and that collections across all institutions were easily searchable.

Although Freezerworks enabled investigators to track samples alongside limited clinical data, it was not integrated with institutional electronic medical record systems, necessitating manual data entry. Investigators recorded basic patient information, including signalment and diagnosis, linked to each sample; however, more comprehensive clinical data, including treatment response and patient outcome, were not systematically captured. This information can only be retrieved through individual case review. This limitation underscores a broader challenge in veterinary biobanking that has been discussed in the biobanking literature.^1^ There is substantial heterogeneity in electronic medical record platforms, coding systems, and clinical terminology across institutions, which hinders efficient integration of clinical data with biobanking infrastructure both within and across sites.

Biospecimen quality control encompasses a broad range of assessments, including assays deigned to evaluate centrifugation delay, exposure to RT, or storage conditions. Our quality control assessment aimed to evaluate prolonged RT exposure and storage conditions across biological fluid type as varying delays between sample collection and storage are inherent to routine clinical practice. It has been established that prolonged storage of human serum at elevated temperatures results in decreased CD40L concentration independent of patient disease status.^15^ Serum samples stored for 48 hours or longer at 20 °C had CD40L concentrations below 4.3 ng/mL, whereas storage for 12 hours or longer at 37 °C resulted in CD40L concentrations below 2.2 ng/mL.^15^ In our study, CD40L concentrations were comparable across institutions and ranged from 3.9 to 24.6 ng/mL. A single sample measured below 4.3 ng/mL. Analysis of the log chart revealed that this sample was stored at 20 °C for 24 hours before transfer to long-term storage, demonstrating that canine CD40L, like human CD40L, is labile at RT. To evaluate urine specimen quality control, we measured TNF-α, a volatile cytokine that is undetectable in human urine after a 30-minute preprocessing delay.^22^ Canine urine TNF-α concentrations in our study ranged from 3.64 to 26.6 ng/mL, indicating appropriate processing for samples in our study. However, human reference values are much lower (0.1 ± 0.2 ng/mL),^23^ supporting the need for quality control biomarker validation in veterinary species.

Careful consideration should be given to quality control biomarker selection and validation in veterinary species. The ability to detect a specific molecule in biological fluid is not necessarily equivalent to a quality sample. We have previously reported that phosphorylated neurofilament-heavy concentration increases with prolonged RT exposure.^24^ Moreover, there is evidence that the specific biomarkers recommended by the ISBER and evaluated in this study are elevated in specific disease conditions, including several neurological diseases. Increased serum CD40L has been reported in human neurological diseases, including traumatic brain injury^25^ and neuromyelitis optica.^26^ Similarly, urine TNF-α concentrations are increased in several human diseases, including chronic low back pain^27^ and acute pyelonephritis.^28^

In recent years, the clinical utilization of blood-based biomarkers has changed the landscape of patient care in human neurodegenerative disease. Fully automated, ultra-sensitive assays that measure biomarkers have become mainstays in human clinical studies of neurodegenerative diseases, including GFAP^29,30^ and Aβ42:Aβ40 ratio.^31^ Diagnostic blood tests for Alzheimer disease biomarkers have recently received FDA approval. Soluble Aβ fragments and derived Aβ42:Aβ40 ratio are indicative of Alzheimer disease–specific neurodegeneration, whereas NfL has been utilized as a diagnostic and therapeutic biomarker for a broader range of neurologic diseases, including Alzheimer disease,^32,33^ amyotrophic lateral sclerosis,^34–36^ and several other neurological diseases. Evaluating these markers as a multiplex panel may provide broader insight into the pathobiology that drives clinical symptoms as many dementia-causing diseases can exhibit similar symptoms but can have distinct effects on biomarker levels. Although less is known about the stability of these proteins in canine samples, NfL has shown some diagnostic utility in veterinary neurological diseases, including canine cognitive dysfunction^18^ and structural epilepsy.^37^ Here, we have validated fitness for purpose of our sample collection, underscoring promising avenues for future biomarker validation that will propel earlier disease diagnosis, improved monitoring of disease progression, and more reliable indicators of treatment efficacy in veterinary patients with neurologic disease.

The authors acknowledge limitations in this study. The survey results reflect response rates ranging from 20% (UC-Davis) to more than 60% (UW-Madison) of faculty, raising the possibility of self-selection bias favoring individuals with a particular interest in veterinary biospecimens. As a result, the desire for institutional resource allocation to support centralized biobanking may be overrepresented and may not reflect the perspectives of the broader faculty or consensus on institutional resource utilization. Additionally, quality control biomarker validation has not been established for veterinary species. In recognition of this field-wide limitation, we evaluated biomarkers recommended by ISBER for human biological fluids. Although our results were broadly consistent with published human data, some discrepancies were observed. While these differences could indicate compromised sample quality, the reproducibility of biomarker concentrations suggests that reference ranges for quality control biomarkers are species specific. These findings highlight the need for systematic validation of quality control biomarkers in veterinary biospecimens and provide rationale for future studies aimed at establishing species-appropriate reference standards.

In conclusion, our data have demonstrated that a shared repository registry is aligned with faculty needs as well as feasible and productive. Multi-institutional veterinary biobanking has the potential to connect veterinarians, physicians, and scientists across basic, translational, and clinical research, enabling rigorous, well-powered multicenter studies that accelerate scientific discovery and ultimately advance both animal and human health.

## Supplementary Material

Supplementary Figure S1

Supplementary Table S1

Supplementary Material S1

Supplementary Material S2

Supplementary materials are posted online at the journal website: avmajournals.avma.org.
