# Supplementary material for: Establishing a standardized biorepository across 3 clinical and translational science center–affiliated veterinary institutions is feasible and productive
Source: Am J Vet Res. Author manuscript; Available in PMC 2026 Aug 13. (PMC13472239; doi:10.2460/ajvr.26.02.0045)

Supplementary Figure S1. Technical duplicates are closely associated across all 4 proteins measured in the Simoa Neurology 4-plex E panel. Selected serum samples (see Figure 5, case 2 and case 3) were re-tested after an additional 1-2 freeze/thaw cycles. Duplicates are close together wherein the percent coefficient of variance (% CV) of the concentration for NfL was 15% and 1%, for Aβ42 10% and 3%, for Aβ40 3% and 4%, and for GFAP 42% (Case 2 only).

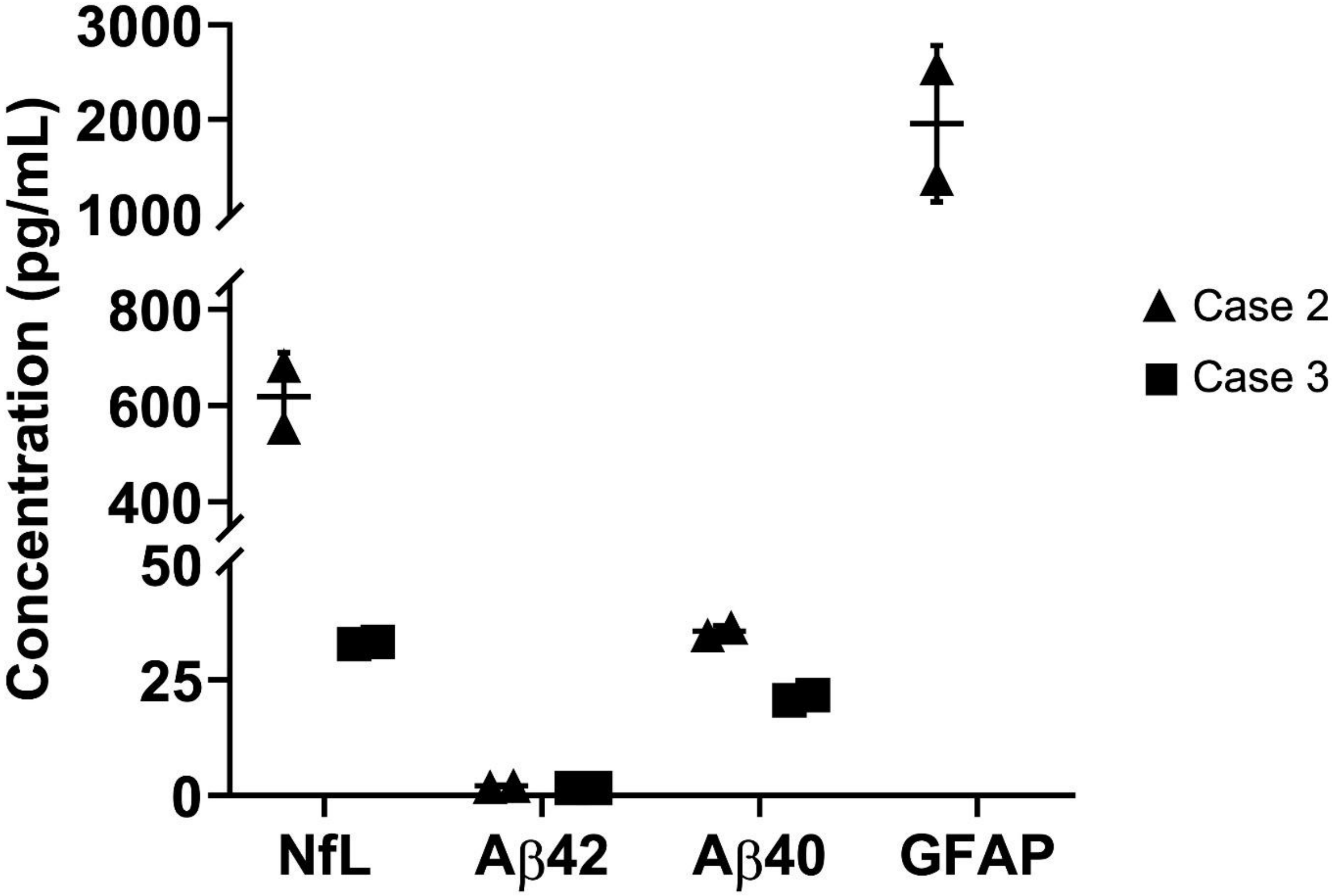

Supplement: Supplementary file 1 [file NIHMS2201979-supplement-Supplementary_Figure_S1.pdf]
