# Supplementary material for: Establishing a standardized biorepository across 3 clinical and translational science center–affiliated veterinary institutions is feasible and productive
Source: Am J Vet Res. Author manuscript; Available in PMC 2026 Aug 13. (PMC13472239; doi:10.2460/ajvr.26.02.0045)
Supplement: Supplementary file 2 [file NIHMS2201979-supplement-Supplementary_Material_S1.pdf]

Supplementary Material S1

Biobanking Interest Survey

We are reaching out as part of a research study to gauge the interest of our research community in establishing a centralized, multi-specialty biorepository.

This survey should take no longer than 4 minutes. No matter your level of interest, your time and efforts are greatly appreciated, as it will help with our research study.

X→

Q1 Does your research currently include work with biological samples from veterinary patients?

- ○ Yes (1)
- ○ No (0)

If you answered NO, Skip To Q12

X→ X→

Q2 Which of following scenarios best applies to your research:

○ I am currently using biological samples from veterinary patients in my research, but my productivity would be enhanced with improved collection, annotation, and storage capacity. (1)

○ I am currently using biological samples from veterinary patients in my research and my system of collection, annotation, and storage is working well. (2)

○ Other: briefly explain (3) ____

X→ X→

Q3 Which type(s) of biological samples do you currently use? (select all that apply)

▢ fresh frozen tissue (1)

▢ FFPE tissue (2)

▢ urine (3)

▢ feces (4)

▢ blood (5)

▢ other: Please specify. (6)
____

X→

Q4 Do you currently have access to a long-term storage space or facility for biological samples?

○ Yes (1)

○ No (0)

Display This Question:

If Q4 = Yes

Q5 Who currently manages your biological sample collection and storage? (Select all that apply)

- ☐ Research Specialist / Technician (1)
- ☐ Undergraduate student(s) (2)
- ☐ Veterinary student(s) (3)
- ☐ Graduate student(s) or resident(s) (4)
- ☐ Post-doc(s) or fellow(s) (5)
- ☐ Myself: Please indicate your title. (6) ________________
- ☐ Other: Please specify. (7) ________________

X→

Q6 Would you be interested in a centralized biobanking service that would collect biological samples from clinical patients, annotate the collections with appropriate clinical information, and maintain a centralized storage facility. Clinicians and other researchers would have access to the samples by request.

- ○ Yes (1)
- ○ No (0)

If you answered NO, Skip To Q12

X→ X→

Q7 Which of the following best describes your interest in a centralized UWVC biobanking service that would collect biological samples from UWVC patients, annotate the collections with appropriate clinical information, and maintain a centralized storage facility. UWVC clinicians and other SVM researchers would have access to the samples by request.

- ○ I support the idea of this service, but I would not require it for my own research. (1)
- ○ I would occasionally use this service for my own research. (2)
- ○ This type of service would be very useful to assist with my research. (3)
- ○ Other: briefly describe. (4) ____________________

Q8 Do you have a collection of biospecimens that you would be willing to contribute to a biorepository for shared use by other individuals?

- ○ Yes (1)
- ○ No (0)
- ○ Yes, but only after my main project is complete (e.g. after an embargo period) (2)
- ○ Other: Please specify. (3) ____________________

Q9 Would you be willing to share biorepository specimens or data with individuals outside of your institution as contribution to collaborative multi-institutional efforts for current or future specimens?

- Yes (1)
- No (0)
- Yes, but only after my main project is complete (e.g. after an embargo period) (2)
- Other: Please specify. (3) ________________

Q10 In your opinion, financial support to sustain biorepository operations should come from:

- Investigator-obtained grant funding contributions (1)
- Institutional support (2)
- Combination (3)
- Another source: Please specify. (4) ________________

Q11 My research would benefit most from samples collected from patients that are seen by the following services or subspecialties (select all that apply).

- ☐ Acupuncture (2)
- ☐ Ambulatory - Equine (75)
- ☐ Ambulatory - Food Animal (76)
- ☐ Ambulatory - Shelter Medicine (77)
- ☐ Anatomic Pathology - Biopsy Service (5)
- ☐ Anatomic Pathology - Necropsy Service (54)
- ☐ Anesthesia (62)
- ☐ Cardiology (7)
- ☐ Clinical Pathology (8)
- ☐ Community Practice (9)
- ☐ Dairy Skills (71)
- ☐ Diagnostic Imaging / Radiology (57)
- ☐ Dentistry & Oromaxillofacial Surgery (12)
- ☐ Dermatology (13)
- ☐ Equine Dentistry (73)
- ☐ Equine Emergency Surgery (14)
- ☐ Equine Internal Medicine (16)
- ☐ Equine Sports Medicine (68)
- ☐ Equine Foal Neonatal Care (69)

- ☐ Equine Ophthalmology (74)
- ☐ Equine Surgery and Lameness (18)
- ☐ Equine Farrier Service (67)
- ☐ Food Animal Production Medicine (19)
- ☐ Food Animal Surgery (72)
- ☐ Large Animal Anesthesia (28)
- ☐ Large Animal Medicine (82)
- ☐ Large Animal Surgery (59)
- ☐ Large Animal Radiology/Ultrasonography (29)
- ☐ Medical Oncology (33)
- ☐ Neurology / Neurosurgery (31)
- ☐ Nutrition (32)
- ☐ Ophthalmology (34)
- ☐ Pharmacy (66)
- ☐ Physical Rehabilitation (70)
- ☐ Primary Care (36)
- ☐ Radiation Oncology (38)
- ☐ Shelter Medicine / Surgery (39)
- ☐ Small Animal Anesthesia (41)
- ☐ Small Animal Critical Care (81)

- [ ] Small Animal Emergency (42)
- [ ] Small Animal Internal Medicine (43)
- [ ] Small Animal Orthopedic Surgery (35)
- [ ] Small Animal Radiology (46)
- [ ] Small Animal Soft Tissue Surgery (47)
- [ ] Special Species (65)
- [ ] WisCARES (63)
- [ ] Wildlife Medicine (79)
- [ ] Zoological Medicine (51)
- [ ] Behavior (84)
- [ ] Theriogenology (17)
- [ ] Laboratory Animal Medicine (83)
- [ ] Other (61) ____

Q12 I would categorize myself as a... (select all that apply).

- ▢ Clinical Track (1)
- ▢ Teaching Track (2)
- ▢ Research Track (3)
- ▢ Assistant Professor (4)
- ▢ Associate Professor (5)
- ▢ Professor (6)
- ▢ Adjunct Faculty (53)
- ▢ Instructor (55)
- ▢ Other (7) ____

Q13 OPTIONAL: If you like, you can provide your email address to be contacted as we follow-up on the survey results and future biobanking endeavors.

____

End of Block: Default Question Block
