# Supplementary material for: Establishing a standardized biorepository across 3 clinical and translational science center–affiliated veterinary institutions is feasible and productive
Source: Am J Vet Res. Author manuscript; Available in PMC 2026 Aug 13. (PMC13472239; doi:10.2460/ajvr.26.02.0045)
Supplement: Supplementary file 3 [file NIHMS2201979-supplement-Supplementary_Material_S2.pdf]

Supplementary Material S2

Neurology & Neurosurgery Sample Handling
Standard Operating Procedure
IACUC #24266

Inclusion criteria:

1. Any neurological patient undergoing advanced diagnostics, surgery, or biopsy.
2. The patient must have a working clinical (presumptive) diagnosis.
3. Consent form must be signed by the client for participation in this study.

Procedure:

1. When collecting biological fluid from a patient (blood, urine, cerebrospinal fluid), please collect 1-mls extra for storage in the Neurology/Neurosurgery Biorepository. Withdrawal from the intravenous catheter is acceptable if venipuncture is not otherwise being performed.

2. There will pre-made 'kits; for serum, urine, CSF and EDTA blood for DNA. Each kit will have a unique Freezerworks ID number, so all tubes in a kit must be used for only 1 patient.

    a. For blood, each kit will contain 4 labeled vials: 3 x **RED** = serum and 1 x **BLUE** for DNA, and an extra label. Label the EDTA tube with the extra label and place in the bag with the empty, labeled **BLUE** vial. The **RED** tubes are for serum and should be frozen.

    b. Urine kits will contain 3 vials for urine = **YELLOW**

    c. CSF kits will contain 3 vials for CSF = **GREEN**.

3. Whole Blood for serum: Collect 1-2 ml of whole blood (intravenously, intravenous catheter scavenge) into a red-top microcentrifuge tube. Place the tube in a rack at room temperature until the blood has clotted (20-30 minutes). Centrifuge the tube at max *g* for 10 minutes. Aspirate the serum and aliquot 500 microliters (0.5 ml) per tube into **RED**-topped T310 cryovials.

4. Whole Blood for DNA: Collect 1 ml of whole blood into an EDTA (purple top) tube. Label and place in bag with 2nd labeled tube and store at 4°C. **DO NOT FREEZE**

5. Urine: Collect 1-2mls of urine (free catch, cystocentesis, or closed collection system). Aliquot 500 microliters (0.5 mls) per tube into **YELLOW**-topped T310 cryovials.

6. CSF: Collect up to 1ml of cerebrospinal fluid into a non-EDTA tube. Aliquot 200 microliters (0.2mls) per tube into **GREEN**-topped T310 cryovials.

7. Record each sample in the sample collection log. Record the patient signalment and presumptive disease. Record the date and time of collection, as well as the sample type and method of collection (e.g., closed collection system for urine). For each kit used, record the Freezerworks ID (JUH ID), sample type, and number of aliquots filled.

8. One the sample log is complete, samples should be transferred to -20°C freezer immediately and the time of storage recorded.

9. Samples and the corresponding logs will be retrieved on a weekly basis and stored at -80°C until further processing.
