# Supplementary material for: Establishing a standardized biorepository across 3 clinical and translational science center–affiliated veterinary institutions is feasible and productive
Source: Am J Vet Res. Author manuscript; Available in PMC 2026 Aug 13. (PMC13472239; doi:10.2460/ajvr.26.02.0045)
Supplement: Supplementary file 4 [file NIHMS2201979-supplement-Supplementary_Table_S1.pdf]

| Institution | Breed | DOB | Age at collection (years) | Sex | Notes |
|---|---|---|---|---|---|
| UPenn | Mixed Breed | 12/25/2015 | 6.5 | MC | |
| UPenn | French Bulldog | 23/20/2018 | 4 | MC | No Urine |
| UPenn | Golden Retriever | 1/7/2014 | 9 | MC | No Urine |
| UPenn | Australian Shepherd | 7/18/2012 | 9 | FS | No Urine |
| | | | | | |
| UC Davis | Shepherd Mix | 8/8/2019 | 3 | MC | |
| UC Davis | Golden Retriever | 11/23/2019 | 3 | F | |
| UC Davis | Labrador Retriever | 7/31/2019 | 3 | MC | |
| UC Davis | Labrador Retriever | 11/23/18 | 4 | MC | |
| | | | | | |
| UW-Madison | Labrador Retriever | 12/7/2015 | 7 | MC | |
| UW-Madison | Alaskan Husky Mix | 2/11/2019 | 3 | MC | |
| UW-Madison | German Shorthaired Pointer | 12/5/2016 | 6 | MC | |
| UW-Madison | Belgian Malinois | 12/12/2017 | 5 | M | |

**Supplemental Table S1.** Case information for samples used for quality control evaluation.
